# Diffuse reduction of cerebral grey matter volumes in Erdheim-Chester disease

**DOI:** 10.1186/s13023-016-0490-3

**Published:** 2016-08-02

**Authors:** Eli L. Diamond, Vaios Hatzoglou, Sneha Patel, Omar Abdel-Wahab, Raajit Rampal, David M. Hyman, Andrei I. Holodny, Ashish Raj

**Affiliations:** 1 Department of Neurology, Memorial Sloan Kettering Cancer Center, 160 E. 53rd. St. Second Floor Neurology, New York, NY 10022 USA; 2 Department of Radiology, Memorial Sloan Kettering Cancer Center, New York, USA; 3 Department of Radiology, Well Cornell Medical College, New York, USA; 4 Human Oncology and Pathogenesis Program, Department of Medicine, Memorial Sloan Kettering CancerCenter, New York, USA

**Keywords:** Histiocytosis, Erdheim-Chester disease, Brain, MRI

## Abstract

**Background:**

Erdheim-Chester disease (ECD) is a rare non-Langerhans histiocytosis characterized by systemic inflammation and granulomatous infiltration of multiple organs including the central nervous system (CNS), bones, and retroperitoneum. CNS infiltration occurs in one third of patients, but cognitive changes are common in patients without CNS disease. Here we investigate whether there is a neuroanatomic basis to observed cognitive deficits, even in absence of CNS disease.

**Methods:**

We present a volumetric analysis of eleven ECD patients without CNS tumors or prior neurotoxic treatments.

**Results:**

Compared to age-matched controls, ECD patients have diffuse, bihemispheric reduction in cortical thickness and subcortical gray matter.

**Conclusions:**

These findings provide the first corroborating evidence for neurologic disease in ECD patients without direct CNS infiltration.

## Background

Erdheim-Chester disease (ECD) is a rare non-Langerhans histiocytosis reported in approximately 550 cases since its initial description in 1930. ECD is a multisystem disease characterized by lipogranulomatous histiocytic infiltration in virtually any organ, although the most commonly affected sites include the bones of the legs, retroperitoneum, heart, orbits, skin, and brain [1]. Historically, ECD was postulated to be an autoimmune-granulomatous disorder characterized by chronic inflammation and cytokine perturbations [2]; recently it has been recognized as an inflammatory myeloid neoplasia associated with oncogenic mutations of kinase signaling including *BRAF*, *NRAS*, *KRAS*, MAP2K1, and *PIK3CA* [1, 3, 4].

Histiocytic infiltrates of the brain or surrounding structures occur in one-third of patients and central nervous system (CNS) involvement is a chief cause of death in ECD [1]. However, neurologic symptoms and signs, including cognitive decline and behavioral changes, have been informally observed in ECD patients without evidence of infiltrative tumors in the brain. These phenomena are of pressing significance to patients but have yet to be characterized clinically or radiographically. As a first attempt to describe neuroanatomical abnormalities in ECD unrelated to neoplastic infiltration, we performed exploratory whole-brain cortical thickness analysis of eleven ECD patients without cerebral CNS lesions or prior cytotoxic chemotherapies and compared them to age-matched controls. Our hypothesis was that there would be reduced cortical thickness in ECD as compared to controls.

## Methods

Eleven patients with histologically confirmed ECD were studied. To eliminate confounding effects of disease or treatment, patients had neither (1) supratentorial lesions on T2-weighted/FLAIR or T1-weighted MRI nor (2) prior cytotoxic chemotherapy. All patients had high-resolution 3D-volumetric T1-weighted gadolinium-enhanced scans performed for standard clinical evaluation. These images were performed on 1.5 T or 3 T scanners (Signa HDxt/Excite, Discovery 450/750, GE Healthcare) using an 8-channel head coil with slice thickness ranging from 1 to 2 mm, in plane resolutions ranging from 0.55 to 1.26 mm^2^ and voxel volumes ranging from 0.74 to 2.04 mm^3^. Images were analyzed with the FreeSurfer (http://freesurfer.net) semi-automated processing pipeline [5, 6]. Images were morphed to Talairach space, white matter intensities were normalized, and the brain portion was extracted by skull-stripping. Because of gadolinium enhancement of the dura and choroid, a custom pre-processing pipeline involving a custom brain mask was developed and added to Freesurfer [7]. SPM’s DARTEL tool was used for a more robust classification of the brain into six tissue types: gray matter, white matter, cerebrospinal fluid, skull, soft non-brain tissue, and air. FSL and AFNI were used to combine these tissue maps and to dilate them to remove hyperintense dura and choroid. This augmented brain mask was then entered into Freesurfer and subsequent steps followed the automated processing stream. An experienced board-certified neuroradiologist inspected and approved all segmentations. Three patients were excluded because of inadequate segmentation. For the purpose of obtaining statistical parameter maps, 14 healthy age-matched subjects’ MRIs were chosen from an ongoing volumetric study of healthy subjects. Acquisition and processing protocols were identical to the ECD group, save that these had fully standardized image acquisitions and did not require custom pre-processing. Group-level whole-brain comparison of cortical thickness and subcortical volumes were performed in the ECD patients and controls. Significance of identified clusters was thresholded at *p* < 0.001 after correction for multiple comparisons with the false discovery rate (FDR) method.

## Results

Seven patients were men and age ranged 48–75 (Table 1). Sites of disease involvement for the entire cohort includes bones, retroperitoneum, mesentery, orbits, subcutaneous and spinal soft tissues, lungs, heart, skin, and posterior fossa. Seven patients had cognitive complaints of inattention or memory difficulties and described compromised performance at work or inability to work entirely. Cerebrospinal fluid was obtained and unremarkable in five patients. Non-specific inflammatory markers were elevated in all patients tested. 10/11 patients had had no ECD treatment, and one was treated with immunosuppression.

**Table 1:** Patient characteristics

Patient	Organ systems affected by ECD	Co-morbid illnesses	Cognitive complaints	Concurrent Medications	Inflammatory Markers^a^	CSF Examination^b^
66 Female	Myocardium, bones	None	No	None	ESR >100	1 WBC; Protein 28; Glucose 57;
					CRP 4.39	
75 Male	Mesentery, orbit, bones, skin	Chronic myelomonocytic leukemia	Yes	Prednisone	CRP 0.98	-
54 Male	Brainstem, retroperitoneum, bones	Diabetes mellitus	No	Prednisolone	ESR 31	2 WBC; Protein 42; Glucose 78
					CRP 1.8	
51 Male	Retroperitoneum, aorta, pericardium, pleura, bones	Essential thrombocytosis	Yes	Interferon-alpha, anakinra	-	1 WBC; Protein 21; Glucose 54
51 Male	Orbit, retroperitoneum, epidural soft tissues, bones	Ulcerative colitis	Yes	None	CRP 1.63	-
60 Male	Retroperitoneum, lung, skin, bones	MALT lymphoma	Yes	Prednisone	ESR 92	2 WBC; Protein 33; Glucose 58
					CRP 1.49	
48 Female	Orbit, retroperitoneum, bones	None	Yes	Prednisone	CRP 3.81	-
66 Female	Brainstem, retroperitoneum, bones	None	Yes	Prednisone	-	2 WBC; Protein 71; Glucose 57
68 Male	Retroperitoneum, bones, subcutaneous soft tissues	None	No	None	ESR 61	-
					CRP 6.29	
58 Male	Orbit, retroperitoneum, bones	None	No	Prednisone	CRP 3.42	-
52 Female	Subcutaneous soft tissues, maxillary sinus, bones, skin	None	Yes	None	CRP 2.56	-

^a^Normal range for ESR 0-15 mm/h; Normal range for C-Reactive Protein <0.80 mg/dl

^b^Normal WBC count in CSF <4 cells per μL; Normal CSF protein 21–38 mg/dL; Normal CSF glucose 38–82 mg/dL

**Fig. 1:**
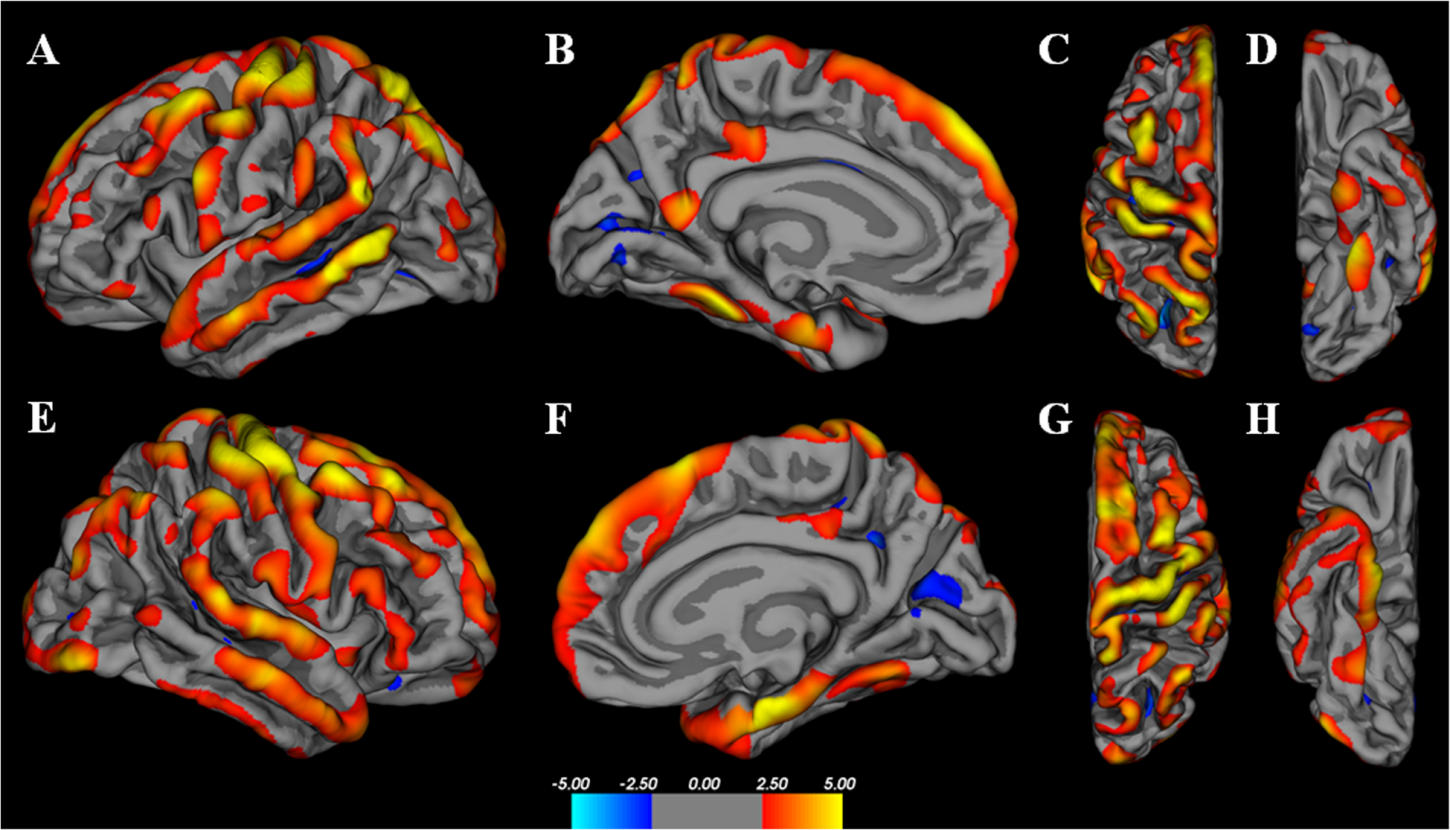
Whole-brain cortical thickness analysis of 11 ECD patients as compared to age-matched controls. Color maps represent statistical significance (*t*-statistic), with yellow representing the greatest statistical significance. Both cerebral hemispheres are represented in lateral projections (**a**,**e**), medial projections (**b**,**f**), superior (**c**,**g**), and inferior (**d**,**h**). The maps demonstrate diffuse and widespread regions of cortical volume loss with a predominance of the frontal and temporal lobes

Comparison of cortical thickness between ECD patients and age-matched controls revealed diffuse bihemispheric reduction in cortical thickness (Fig. 1). Analyses were performed including and excluding the two patients with infratentorial disease with entirely comparable results; therefore, the findings of all 11 patients are presented. After FDR correction, 37 clusters were significant at *p* < 0.001, 21 in the left hemisphere and 16 in the right (Table 2). Of these, 17 were in parietal cortices (9 left, 8 right), 10 were in frontal cortices (6 left, 4 right), 6 were in temporal cortices (3 left, 3 right), and 4 were in occipital cortices (3 left, 1 right). The largest clusters (greater than 10 cm^2^ surface area on the Desikan-Killarney standard brain atlas) were in the right precentral gyrus (38.32 cm^2^), right superior frontal gyrus (38.56 cm^2^), left supramarginal gyrus (33.59 cm^2^), left superior frontal gyrus (19.86 cm^2^), left precentral gyrus (19.42 cm^2^), right entorhinal cortex (16.62 cm^2^), and left superior parietal gyrus (10.52 cm^2^). Total subcortical grey matter volume was significantly reduced in ECD patients (mean volume 57 cm^3^ ± 5.1) compared to controls (158 cm^3^ ± 21.6; *p* < 0.0001; Table 2). There were no differences in white matter or cerebellar volumes between patients and controls.

**Table 2:** Comparison of cortical thickness and subcortical volumes between ECD Patients and age-matched controls

A. *Left Hemisphere*							B. *Right Hemisphere*						
Cluster	Location	Size (mm2)	logP	TalX	TalY	TalZ	Cluster	Location	Size (mm2)	logP	TalX	TalY	TalZ
1	Superior parietal	1052	−7.81	−19	−65	59	1	Precentral	3832	−7.06	42	−6	55
2	Supramarginal	3359	−7.62	−59	−51	21	2	Postcentral	601	−6.42	44	−28	62
3	Postcentral	602	−7.35	−42	−29	63	3	Entorhinal	1662	−6.00	21	−10	−30
4	Precentral	1942	−6.64	−29	−16	67	4	Superior frontal	3856	−5.62	9	20	60
5	Superior frontal	1986	−6.39	−7	50	40	5	Precentral	107	4.96	19	−31	55
6	Inferior parietal	539	−6.06	−35	−77	38	6	Superior temporal	884	−4.83	66	−32	10
7	Caudal middle frontal	915	−5.66	−35	16	51	7	Supramarginal	443	−4.81	57	−33	45
8	Fusiform	313	−5.26	−30	−40	−22	8	Lateral occipital	920	−4.68	43	−83	−12
9	Inferior parietal	417	5.20	−32	−74	20	9	Inferior parietal	870	−4.67	46	−60	45
10	Precentral	277	−5.13	−58	6	25	10	Superior parietal	280	−4.15	34	−49	63
11	Precuneus	216	−4.25	−7	−55	16	11	Superior parietal	660	−4.07	21	−78	44
12	Entorhinal	207	−4.13	−20	−10	−31	12	Fusiform	316	−3.71	29	−57	−15
13	Posterior cingulate	261	−3.61	−6	−29	38	13	Inferior parietal	266	3.17	33	−70	23
14	Pericalcarine	489	3.58	−9	−82	12	14	Precuneus	79	3.16	12	−54	33
15	Lateral occipital	375	−3.51	−16	−100	5	15	Pars opercularis	464	−3.03	55	22	16
16	Supramarginal	195	−3.42	−61	−31	36	16	Posterior cingulate	158	−3.02	6	−19	40
17	Inferior temporal	113	−3.28	−46	−12	−39							
18	Lateral occipital	98	−3.24	−43	−82	0							
19	Lateral occipital	101	−3.04	−34	−87	10							
20	Precentral	122	−3.01	−57	2	7							
21	Pars orbitalis	68	−3.01	−46	36	−14							
C. *Subcortical Volumes*													
Volume				Group		Mean volume (cm3)		STD		95 % CI		*p*-value	
Subcortical gray matter (all)				ECD		57		5.1		5.4–6.1		<0.0001	
				Control		158		21.6		145.7–170.6			
White matter (right hemisphere)				ECD		247		42.3		218.8–275.7		0.75	
				Control		237		388		214.0–258.9			
White matter (left hemisphere)				ECD		246		43.5		216.3–274.7		0.74	
				Control		238		39.7		214.3–260.2			
Cerebellar grey matter (right)				ECD		50		6.9		45.0–54.2		0.6	
				Control		43		8.1		38.1–47.5			
Cerebellar grey matter (left)				ECD		48		7.4		42.8–52.7		0.97	
				Control		43		7.5		38.9–47.6			

Significant clusters with FDR corrected *p* <0.001 are listed in order of descending statistical significance with anatomic location, cluster size in mm^2^, log(10)P, and Talairach coordinates for the cortical regions of the left (A) and right (B) hemispheres. Comparison of subcortical volumes, including total white matter, total grey matter, and total cerebellar volumes, is presented in (C)

## Discussion

In this study we present group-level analysis of cortical thickness and subcortical volumes in 11 ECD patients compared to age-matched healthy controls. Despite the small number of patients, reduction of cortical thickness and subcortical grey matter volumes in ECD patients was demonstrated to a statistical significance of *p* < 0.001 in 37 cortical clusters after correction for multiple comparisons. These findings suggest the possibility of a diffuse and significant neurodegenerative process at hand. This study provides the first objective evidence to corroborate a common clinical observation of neurologic dysfunction in ECD patients without cerebral tumors. Further confirmation and characterization of this process in its relation to ECD is vital not only to envision potential interventions but also to advocate for appropriate medical and disability benefits.

Neuropathologic studies of ECD are sparse and describe mass lesions whose histopathology reveals classic histiocytic infiltrates with admixed inflammation [8]. No pathologic studies of brains ostensibly unaffected by ECD have been performed. There is greater literature about neurologic and neuropathologic findings in Langerhans cell histiocytosis (LCH), an entity closely related to ECD. Non-infiltrative neurodegenerative phenomena are recognized in pediatric LCH, although rare. The spectrum of findings includes T2 abnormalities in the cerebellum, cerebellar degeneration, supratentorial leukoencephalopathy, dilation of Virchow-Robin spaces, and rarely diffuse cerebral atrophy [9]. Subclinical neurocognitive deficits have been found in a minority of long-term LCH survivors, although without correlation to neuropathologic findings, and it remains unclear whether these are sequelae of disease or treatment [10]. Mechanisms underlying non-infiltrative disease in LCH have not been identified, although paraneoplasia has been postulated and modest therapeutic benefit has been seen with immunosuppressive and cytotoxic therapies [9].

Further study is necessary to suggest a mechanism for reduction in cerebral volumes in ECD. Our study does not establish whether this phenomenon is restricted to grey matter, as we did not examine the white matter in a dedicated fashion. It is possible that reduction in grey matter is secondary to a primary process involving the white matter, such as has been postulated in subcortical dementias [11, 12]; in our patients there is no visible leukoencephalopathy, but inapparent white matter dysfunction is possible. Also, it is worth noting that the diffuse pattern of grey matter loss is different from that seen with other neurodegenerative entities, such as Alzheimer’s disease, Lewy body dementia, and HIV-associated neurodegeneration, which have various regional distributions [13–15]. Because ECD can be diagnosed years and even decades after first symptoms, the possible chronicity of disease in these patients suggests that grey matter changes may have been more regional and less diffuse if measured earlier. Prospective and longitudinal study of this nature in ECD patients would be extremely informative.

A defining feature of ECD is robust, chronic, and uncontrolled systemic inflammation. Because of its extreme rarity, ECD is frequently diagnosed after several years of symptoms, and therefore inflammation is longstanding even in newly diagnosed patients. Studies have described a signature pattern of cytokine perturbations in ECD, including elevations in TNF-α, IL-1β, and IL-6, and it has been shown that a cytokinemic state persists even in the setting of treated disease [2, 16–18]. Systemic inflammation itself has been implicated as a cause of neurotoxicity and neurodegeneration [19–21], and it is plausible that such a process is operative in ECD. One potential pathogenic mechanism involves cytokines that readily pass the blood-brain barrier, activate microglia, and perpetuate further maladaptive inflammatory events [19]. It is notable that the cytokines imputed in this process, TNF-α, IL-1β, and IL-6, are specifically those elevated in ECD. Cytokine studies of CSF have not been performed to corroborate this idea or to provide any evidence of neuroinflammation in ECD.

There are several limitations to this exploratory study. First, our cohort is small. Furthermore, there are no neurocognitive data to quantitate cognitive complaints or to identify subclinical deficits. The ulta-rarity of this orphan disease, however, renders it uniquely difficult to collect data prospectively. In light of this, a sample of 11 evaluable high-resolution images of patients without confounding treatments represents a relatively large cohort without precedent. Regarding image processing, scans were obtained for clinical care, and therefore there was variability in acquisition parameters and magnet strength. For that reason, scans were carefully screened and removed if segmentation quality precluded accurate volumetrics. Furthermore, cortical thickness measurement by Freesurfer has demonstrated consistency across scanning platforms and field strengths [22]. In addition, the uniformity of our findings and their statistical strength suggests they will be reproduced in larger studies. Finally, it should be noted that several patients were taking corticosteroids, and this has been independently found to be associated with brain atrophy by a variety of possible mechanisms including osmotic effects [23]. This finding has been observed mainly in the context of high-dose steroids or long-term low-dose steroids, neither of which was administered to our patients. Confirmation of these findings in a prospective study with parallel analysis of complementary imaging modalities, assessment of cognitive function, and correlation with markers of serum and CSF inflammation is necessary.

## Abbreviations

CNS, central nervous system; CRP, C-reactive protein; CSF, cerebrospinal fluid; ECD, Erdheim-Chester Disease; ESR, Erythrocyte sedimentation rate; FDR, False discovery rate; MRI, magnetic resonance imaging
